# Blood-derived gene expression profiles associated with dietary microalgae oil intake and methane emission variation in lambs

**DOI:** 10.1186/s12864-026-13110-1

**Published:** 2026-06-23

**Authors:** Steffimol Rose Chacko Kaitholil, Mark H Mooney, Aurélie Aubry, Omar Cristobal-Carballo, Richard Hillis, Vahid Razban, Tianhai Yan, Faisal I Rezwan, Steven Morrison, Sharon Huws, Masoud Shirali

**Affiliations:** 1 Institute for Global Food Security, School of Biological Sciences, Queen’s University, Belfast, BT9 5DL Northern Ireland, UK; 2 Agri-Food and Biosciences Institute (AFBI), Large Park, Hillsborough, County Down BT26 6DR Northern Ireland, UK; 3 School of Medicine, Dentistry and Biomedical Sciences, Queen’s University, 97 Lisburn Rd, Belfast, BT9 7BL Northern Ireland; 4 Department of Computer Science, Aberystwyth University, Aberystwyth, SY23 3DB Wales, UK

**Keywords:** Methane, Transcriptomics, Gene expression, Lipid metabolism, Cholesterol, Sheep, Ruminants, Livestock

## Abstract

**Background:**

Minimising methane (CH_4_) emissions from livestock production is a global priority, and feed modifications, such as supplementing diets with microalgae, have previously been shown to help reducing enteric CH_4_ production. This study explored blood-derived host gene expression profiles from twenty lambs supplemented with increasing levels of microalgae oil to investigate their transcriptional responses associated with varying microalgae oil levels while also exploring the host systemic responses towards varied CH_4_ productions.

**Results:**

Findings revealed no significant changes in CH_4_ production with increasing levels of microalgae oil intake through phenotypic analysis (*P* = 0.18). However inter-individual variations in CH_4_ production ranged from 27.02 to 47.86 g/day throughout the study period. Blood RNA-Sequencing identified 64 significant genes including *DHCR7*, *DHCR24*,* HMGCS1*,* INSIG1*,* LSS*,* MSMO1*, and *SQLE*, which were involved in lipid metabolism, and steroid biosynthesis that became enriched alongside increasing microalgae oil intake levels thereby contributing to a positive impact on lambs’ metabolic functions. Additionally, seven significant blood-expressed host genes (*NME4*,* MARCHF3*,* PLXNB3*, *LOC132657460*,* LOC121819234*,* LOC105603087*,* LOC101116551*) functionally enriched in nucleotide metabolic pathways and immune responses were identified to have significant positive associations with increasing CH_4_ production. Importantly, this study found no overlap between genes associated with microalgae oil intake and those linked to CH_4_ emissions.

**Conclusions:**

Findings suggest that microalgae oil intake and inter-individual variations in CH₄ production are associated with distinct blood-derived transcriptional responses. Although such signals should be interpreted as proxies for systemic host responses rather than direct measures of rumen-specific processes, these results emphasise the importance of considering host-associated molecular variations alongside dietary CH₄-mitigation strategies.

**Supplementary Information:**

The online version contains supplementary material available at https://doi.org/10.1186/s12864-026-13110-1.

## Background

The persistent rise in greenhouse gas (GHG) emissions poses a considerable challenge to climate change mitigation efforts, with methane (CH_4_) from livestock production systems being a particularly significant contributor. Sheep produce CH_4_ as a byproduct of ruminal fermentation, a process involving breakdown of feed by rumen microbes, leading to volatile fatty acids (VFA) production including propionate, acetate, and butyrate along with hydrogen (H_2_) release, which is utilized by methanogens to produce CH_4_ [1]. This process accounts for approximately 15% of overall feed energy intake [2] necessitating increased feed consumption to compensate for energy loss and contributing to further GHG generation during feed production, increasing costs and reducing economic efficiency. Consequently, several diet-based strategies have been explored aiming to reduce and suppress livestock CH_4_ production [3–5].

Microalgae (*Schizochytrium sp*.) has been identified as one of the promising supplements [6] to reduce CH_4_ emissions in vitro [7] and in vivo [8, 9]. However, as reported in sheep and cows [10], excessive microalgae use can negatively affect dry matter intake (DMI) and feed palatability. This underscores the importance of balanced supplementation, which may require long-term trials to assess both effects on animals and the potential of rumen microbes to adapt to such diet manipulation strategies limiting effectiveness. Consequently, it is equally crucial to explore additional approaches alongside dietary modifications, which can further contribute to CH_4_ emission lowering.

The adoption of genetic or genomic selection methods to identify animals with inherently low CH_4_ production offers complimentary avenues for long-term mitigation of CH_4_, given the moderate trait heritability (heritability, *h*^*2*^ = 0.29 ± 0.05) [11]. Transcriptomics-based ribonucleic acid sequencing (RNA-Seq) can support these methods by identifying host genes and pathways associated with variation in CH_4_ productions. However, only few RNA-Seq studies [12, 13] have explored CH_4_ production in sheep particularly using rumen samples, and blood gene expression profiles associated with CH_4_ variations still remain unexplored in sheep and whole livestock, apart from a comparable study in beef-on-dairy cattle [14]. In addition, little is known about host blood transcriptomic responses to varying levels of dietary microalgae oil.

Blood samples can therefore be an important proxy for CH_4_ studies, as CH_4_ production is not only influenced by rumen fermentation, but also by host physiological and metabolic status. While CH_4_ is produced in the rumen, host-associated factors such as genetics, feed intake patterns or metabolism can influence CH_4_ production [15] and subsequently partially reflected in blood RNA profiles [16]. For instance, immune system activities or metabolic stress can indirectly influence CH_4_ production by modifying patterns of feed intake, partitioning of nutrients, rate of digestion as well as functioning of rumen [16]. A recent study on Japanese black cattle has shown correlations between circulating blood metabolites including beta hydroxy butyric acid, and CH₄ production, supporting the relevance of blood-based molecular phenotypes for CH_4_-related traits [17]. In contrast, rumen sampling is an invasive procedure requiring surgical fistulation or tissue collection post-slaughter, making it less practical for studies involving live animals [18]. While blood metabolomics is useful to detect downstream biochemical markers associated with CH_4_ production, blood RNA-Seq may provide additional insights into upstream pathways such as host immune, and physiological responses, indirectly contributing to alterations in CH_4_ production, and thus can serve as a proxy for systemic host responses associated with inter-individual CH_4_ variation and increasing intake levels of microalgae oil.

Therefore, this study used whole blood RNA-Seq in lambs to investigate host transcriptional responses towards graded levels of microalgae oil (*Schizochytrium* sp.) supplementation and inter-individual variations in CH_4_ production. CH_4_ production was chosen as the primary phenotype other than CH_4_ yield or intensity because CH₄ production represents the total amount of CH_4_ produced by an animal. However, CH_4_ yield or intensity are CH_4_ efficiency traits which are ratio-based and incorporate other phenotypic variables (like feed intake) into calculation. Consequently, variations in CH_4_ yield or intensity may arise due to changes in either CH_4_ emissions or denominator trait, making biological interpretation complex [19]. We hypothesize that dietary microalgae oil induces distinct molecular responses in the host and that inter-individual variations in CH_4_ production are associated with specific transcriptional pathways. By linking blood gene expression profiles in lambs with dietary microalgae oil and CH_4_ measurements, this study aimed to characterize host-level transcriptional signatures that may complement nutritional and genetic strategies for CH_4_ mitigation while maintaining animal productivity.

## Materials and methods

### Experimental design and animal management

Samples in this study were obtained from a previously conducted experimental trial [20, 21]. Briefly, twenty male weaned lambs (crosses of Texel and mule (Bluefaced Leiscester x Scottish Blackface breeds), with average age of 13 ± 1.20 weeks (mean ± standard deviation), average weight of 30 ± 3.6 kg) were acquired from Agri-Food and Biosciences Institute (AFBI), Loughgall flock which were then housed and managed at the AFBI Hillsborough research facility. Lambs were assigned to one of the four dietary groups, which consisted of a total mixed ratio (TMR) diet introduced as 50:50 ratio of grass silage and concentrate at dry matter (DM) basis (see Additional file 1). Diets were formulated to be isoenergetic and isoproteic, providing approximately 11.72 MJ/kg DM metabolizable energy and 13.66 gN/kg DM metabolizable protein, supporting a target live weight gain of 0.20 kg/day. Dietary groups differed only in the level of microalgae oil (*Schizochytrium* sp.) included in the concentrate component, specifically: 0.00% (Control, *n* = 6), 0.54% (Low, *n* = 4) 1.08% (Medium, *n* = 5) and 1.62% (High, *n* = 5) per kg DM along with *ad libitum* access to water. To balance the microalgae oil intake across the four dietary groups, soya oil was used at inverse rations: 1.62% (Control, *n* = 6), 1.08% (Low, *n* = 4) 0.54% (Medium, *n* = 5) and 0.00% (High, *n* = 5)). The TMR diet was provided once per day, between 09:00 and 10:00 h. The study was conducted over nine weeks, including a prior three‑week adaptation period during which lambs were housed in individual pens and offered *ad libitum* grass silage. Feed intake was recorded daily, and body weight was measured weekly for each lamb. Dry matter intake (DMI) and average daily live weight gain (ADG) were individually calculated as follows:

- $$\:DMI\:\left(\frac{kg}{day}\right)=\mathrm{A}\mathrm{s}-\mathrm{f}\mathrm{e}\mathrm{d}\:\mathrm{f}\mathrm{e}\mathrm{e}\mathrm{d}\:\mathrm{a}\mathrm{m}\mathrm{o}\mathrm{u}\mathrm{n}\mathrm{t}\:\left(\mathrm{k}\mathrm{g}\right)\times\:\mathrm{D}\mathrm{M}{\%}\:$$, where,
  $$\mathrm{As}-\text{fed feed amount}\,(\mathrm{kg})=\text{feed offered}\,(\mathrm{kg})-\text{feed refused}\,(\mathrm{kg})$$
- $$\:ADG\:\left(\frac{g}{day}\right)=\:\frac{Final\:body\:weight\:\left(kg\right)-initial\:body\:weight\left(kg\right)}{Days\:on\:feed\:\left(9\:weeks\:or\:63\:days\right)}\times\:1000$$

After nine weeks, lambs were moved to indirect open-circuit respiratory chambers for three days following 1 day of adaptation. CH_4_ production (g/day) for each animal was measured over the last 48-hour period of the CH_4_ production measurements.

### Sample acquisition

Blood was collected in the morning following a 3-hour period of fasting and post CH_4_ measurement (on the last day of chamber trial) using PAXgene blood RNA tubes (BD, Franklin Lakes, New Jersey, USA; and QIAGEN, Venlo, Netherlands), centrifuged (2,750 g,10 min at 4 °C) and plasma was isolated. The animal study work was conducted at the Agri-Food and Biosciences Institute (AFBI) Hillsborough, under the regulations of the Department of Health for Northern Ireland, according to the United Kingdom (UK) Animals (Scientific Procedures) Act 1986 requirements and AFBI (Hillsborough) Animal Welfare and Ethical Review Body approval.

### RNA extraction, sequencing and data analysis

Total RNA was isolated from acquired plasma using PAXgene Blood RNA Kits following manufacturer’s protocols. Subsequently, RNA samples underwent library preparation using the TruSeq3 RNA Library Prep Kit v2 (Illumina, San Diego, California, USA). Resulting libraries underwent quality control (QC) to ensure the expected size distribution of cDNA fragments and adapter-ligated libraries were sequenced on the Illumina NovaSeq Platform employing 150 bp (bp) paired-end reads according to the manufacturer’s protocol. To ensure data quality, the sequenced data underwent sample QC using FastQC (v0.12.1) [22] followed by pre-processing steps, such as adapter sequence trimming and filtering for low-quality reads, using Trimmomatic (v0.39) [23]. The trimmed reads were aligned to sheep (*Ovis aries*) reference genome (Genome assembly, ARS-UI_Ramb_v3.0^1^) available in the National Centre for Biotechnology Information (NCBI) using the HISAT2 (v2.1.0) [24] software. A count matrix was generated using featureCounts (v2.0.1) [25] representing the number of mapped reads for each gene. Normalization of the count matrix was performed using DESeq2 [26] which uses the median-of-ratios method to correct for sequencing depth variation. To filter out the genes with low expression, as genes with low counts often reflect non-meaningful associations [27], a strict filtering criterion was applied where genes with counts less than 10 were first converted to zero and subsequently removing all rows with zero counts in more than 70% samples. Total consumption of microalgae oil was adjusted for animals’ body weight (g/kg body weight (BW)) to account for individual differences in body size and feed intake. DGE analyses were conducted using DESeq2 in R (v 4.3.3) [28], incorporating microalgae oil intake (g/kg BW) and CH_4_ production (g/day) as continuous traits, thereby allowing gene expression levels to vary as a linear function of microalgae oil intake and CH_4_ production which were analysed as two separate analyses within the DESeq2 design. Modelling these traits as continuous phenotypes improves statistical power by leveraging the full spectrum of phenotypic variation rather than relying on discrete group contrasts. A primary cut-off threshold was established for the *adjusted P*-value (*P*_adjusted_) from the Wald test using the Benjamini-Hochberg (BH) method and fold change, specifically with *P*_adjusted_ < 0.05 and an absolute Log_2_ fold change (henceforth |Log_2_ fold change|) ≥ 2 (4-fold change) to identify significant differentially expressed genes (DEGs) [29]. To further investigate the relationship between CH_4_-associated gene expression and CH_4_ production, a linear regression analysis was carried out in R utilizing the normalized count matrix obtained from variance stabilising transformation (VST) method in DESeq2. Model outputs generated from the linear regression analyses such as regression coefficient (β), coefficient of determination (R^2^), and associated *P* were extracted. *P* values were then subjected to multiple testing correction using the BH method. Scatter plots were generated to better visualize each gene’s association.

### Functional enrichment analysis of DEGs

To infer biological processes associated with varying levels of microalgae oil and CH_4_ production involving significant DEGs, functional overrepresentation analysis (ORA) were conducted with the R package clusterProfiler [30] based on gene ontology (GO) and Kyoto encyclopedia of genes and genomes (KEGG) databases. As input gene list for DEGs, the list of DEGs ranked for decreasing absolute Log_2_ fold change was used. Analyses were conducted with a BH corrected *P* cutoff of 0.05 applied to identify significant GO terms and KEGG pathways. The AnnotationHub (v 3.12.0) (hub: AH114633) [31] was used to annotate the GO entry for sheep.

### Construction of Protein-Protein Interaction (PPI) network on DEGs associated with microalgae oil inclusion in the diet

For constructing the PPI network from DEGs associated with increased microalgae oil intake, STRING database [32] was used. A cut-off criterion of a combined score greater than 0.4 (medium confidence) and greater than 0.7 (high confidence) was applied to ensure the reliability of the interactions. Subsequently, the outcomes were visualized using CytoScape software v.3.10.1[33]. Highly interconnected (hub) genes in the PPI network were determined by employing CytoHubba, another CytoScape plug-in, based on their degree of association with other genes in the PPI network. Maximal clique centrality (MCC) algorithm in CytoHubba was employed to evaluate and select the top hub genes.

### Gene semantic similarity analysis

Microalgae oil intake-associated and CH_4_-associated DEGs were mapped to their corresponding human orthologs to retrieve their Entrez Gene identifiers (IDs). GO semantic similarity analysis was performed using the GOSemSim R package [34] by employing mgeneSim() function in two separate analyses for each trait-specific gene set utilizing the Wang method and Best-Match Average parameter. Similarity score computation was carried across the three GO categories: Biological Process (BP), Molecular Function (MF), and Cellular Component (CC). For exploratory purposes on CH_4_-associated gene set, a less stringent threshold was applied on the normalized gene set from DESeq2 where CH_4_-associated genes with *P*_adjusted_ < 0.05 and |Log_2_ fold change| > 0 were extracted and gene semantic similarity analyses were carried out. Results were visualized using pheatmap R package [35]. Furthermore, to observe any semantic similarities between microalgae oil-associated gene cluster and CH_4_-associated gene cluster, clusterSim() function was utilised.

### Statistical analysis

Statistical analysis of the data was performed using R software v4.4.2 [28]. To assess normality, the Shapiro-Wilk test was employed. Equality of variances among the groups was evaluated using Bartlett’s test, followed by a One-Way Analysis of Variance (ANOVA) to assess whether different inclusion levels of microalgae oil influenced body weight, DMI or CH_4_ emissions. A cut-off of *P* < 0.05 was used to determine statistically significant effects. Results were presented as means ± standard error of mean (SEM), providing an indication of the variability within the data.

## Results

### Phenotypic responses to microalgae oil inclusion

Table 1 presents a summary of the mean growth performance, DMI, and CH_4_ production in twenty lambs included in this study fed on increasing levels of microalgae oil over a 9-week period. The final body weight of lambs receiving no microalgae oil was slightly higher than that of lambs supplemented with 5.4–16.2 g/kg DM of microalgae oil to replace the same levels of soya oil; however, this difference was not statistically significant (Initial bodyweight: *P* = 0.66, *F* = 0.54; Final bodyweight: *P* = 0.81, *F* = 0.31*)*. Additionally, supplementation of microalgae oil to replace soya oil did not lead to a significant effect on DMI (*P* = 0.62, *F* = 0.60). Although mean CH_4_ production across the four dietary groups varied among studied lambs, ranging from 40.87 ± 2.09 g/day (control) to 34.11 ± 1.59 g/day (high) (mean ± SEM) (Table 1), these variations were also not statistically significant (*P* = 0.18, *F* = 1.86). However, a significant negative correlation between CH_4_ production and actual intake levels of microalgae oil was observed (R^2^ = -0.45, *P* = 0.04*)*, while no significant correlation between CH_4_ production and DMI (R^2^ = 0.33, *P* = 0.15*)*, or final body weight (R^2^ = 0.44, *P* = 0.05).

**Table 1:** Phenotypic effects of microalgae oil inclusion. Mean growth performance, DMI and CH_4_ production in twenty lambs supplemented various levels of microalgae oil to replace the same amount of soya oil are tabulated. The data is presented as mean ± SEM. No significant differences were observed in the phenotypes studied with increasing levels of microalgae oil

Phenotype	0 g/kg DM (control) (*n* = 6) (mean ± SEM)	5.4 g/kg DM (low) (*n* = 4) (mean ± SEM)	10.8 g/kg DM (medium) (*n* = 5) (mean ± SEM)	16.2 g/kg DM (high) (*n* = 5) (mean ± SEM)	*P*
Initial body weight (kg)	35.91 ± 2.09	34.37 ± 2.11	35 ± 1.36	33 ± 0.82	0.66
Final body weight (kg)	50.41 ± 3.22	47.62 ± 3.33	48.20 ± 2.00	47.10 ± 1.91	0.81
DMI (kg/day)	1.30 ± 0.05	1.21 ± 0.07	1.29 ± 0.06	1.23 ± 0.03	0.62
ADG (g/day)	187 ± 25.58	199 ± 12.69	159 ± 13.55	180 ± 16.32	0.57
CH_4_ production (g/day)	40.87 ± 2.09	37.38 ± 2.15	36.24 ± 2.66	34.11 ± 1.59	0.18

### RNA-sequencing

On average, 83.4% (24,668,185 reads) of the total reads aligned uniquely with the sheep reference genome obtained from the NCBI database (see Additional file 2). Furthermore, 72% (44,533,410 reads) of the aligned reads were successfully assigned to gene symbols corresponding to the reference genome build (see Additional file 2), resulting in the identification of 31,304 genes. Subsequently, 8,875 genes were excluded due to low expression levels (gene expression counts < 10 in over 70% of samples) which left a total of 22,429 genes for further downstream analyses, including differential gene expression (DGE) analyses and ORA.

### Increasing levels of microalgae oil influenced the downregulation of genes involved mainly in steroid biosynthesis

**Fig. 1:**
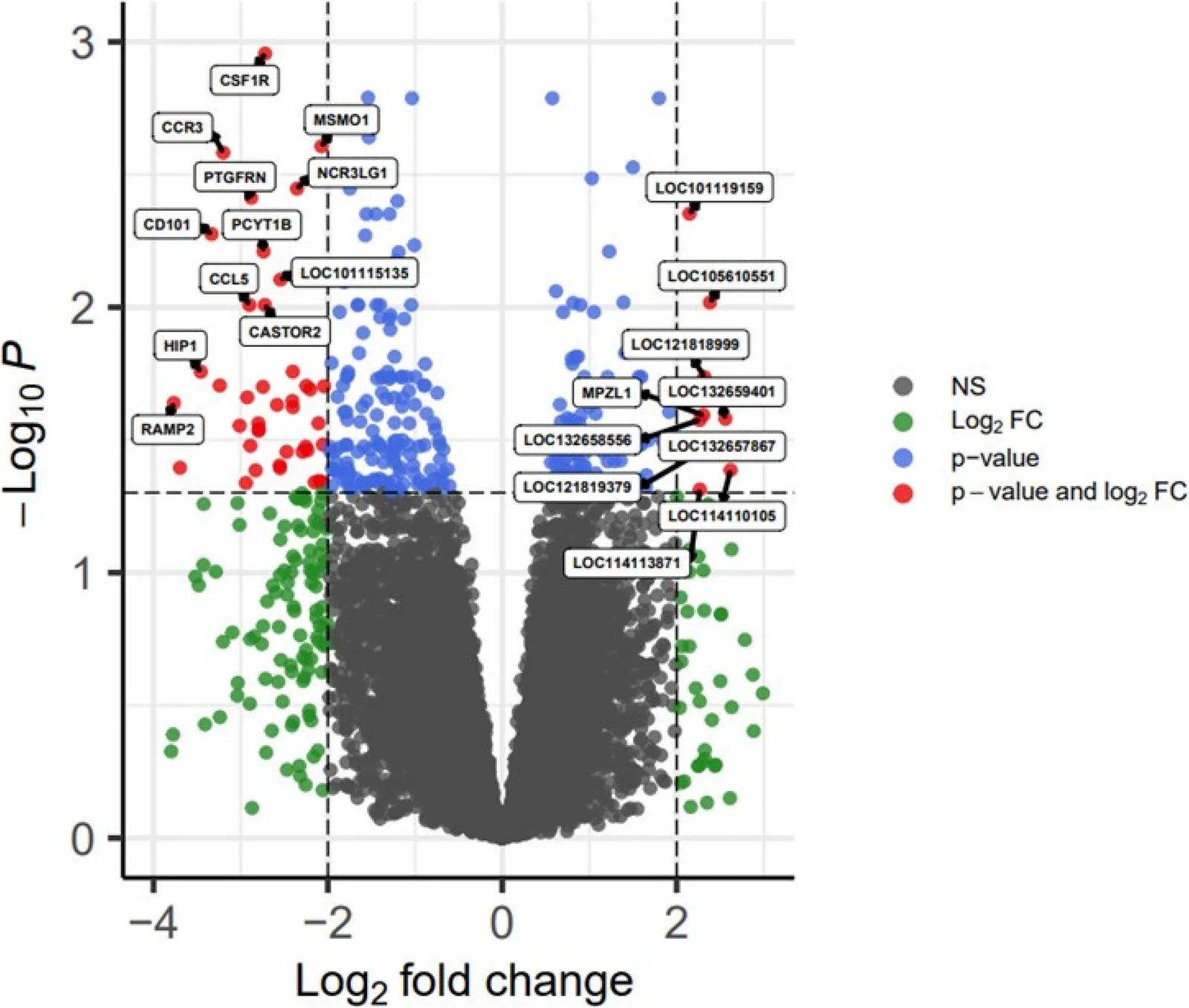
DEGs associated with microalgae oil replacement rates for soya oil (g/kg BW) in lambs. The volcano plot represents Log_2_ fold change on the x-axis and -Log_10_ *P* on the y-axis. Red colour dots represent genes that passed both *P*_*adjusted*_ and |Log_2_ fold change| cutoff. Green dots represent genes that only satisfy |Log_2_ fold change| cutoff, and blue dots represent genes that only passed *P*_*adjusted*_ cutoff. Grey dots represent non-significant (NS) genes

**Fig. 2:**
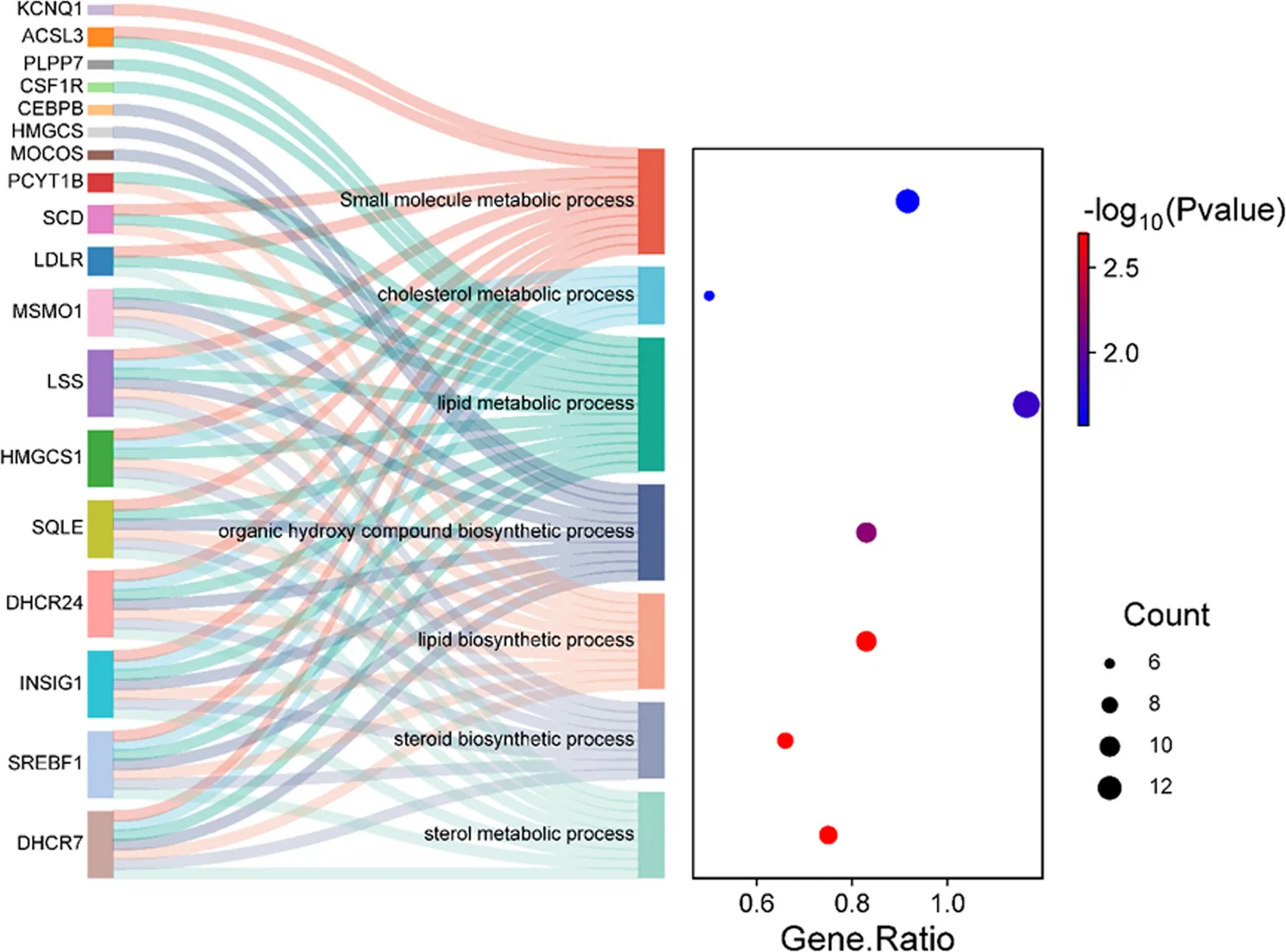
Top enriched pathways of DEGs of microalgae oil replacement rates for soya oil (g/kg BW). The x-axis indicates number of genes (in ratios) associated with each pathway, and the y-axis represents -log_10_ (*P*). Each circle is color-coded according to -log_10_ (*P*), with red indicating the highest significance

DGE analysis revealed a total of 64 DEGs, comprising 12 upregulated and 52 downregulated genes that were statistically significant with increased microalgae oil intake (*P*_*adjusted*_ < 0.05 and |Log_2_ fold change| ≥ 2) (Fig. 1, see Additional file 3). The top five upregulated and downregulated genes are listed in Table 2. ORA was conducted on these DEGs, revealing that they were enriched in GO terms in the BP category, with “sterol metabolism”, “steroid biosynthesis”, and “lipid biosynthesis” identified as the top enriched terms (Fig. 2, see Additional file 4). The KEGG pathway enrichment analysis indicated “steroid biosynthesis” as the only enriched pathway, involving five downregulated genes: 24-dehydrocholesterol reductase (*DHCR24*), 7-dehydrocholesterol reductase (*DHCR7*), methyl sterol monooxygenase 1 (*MSMO1*), lanosterol synthase (*LSS*), and squalene epoxidase (*SQLE*) (see Additional file 4).

**Table 2:** Top DEGs associated with microalgae oil replacement rates for soya oil (g/kg BW) in lambs. The significant threshold was determined as *P*_*adjusted*_ < 0.05, and absolute |Log_2_ fold change| = 2. Microalgae oil intake was treated as a continuous phenotype. Hence Log_2_ fold change here corresponds to fold change per unit increase in microalgae oil intake. The *P*_*adjusted*_ was calculated by adjusting *P-value* from Wald test using BH method

Gene ID	Gene name	Log_2_ fold change	*P*_adjusted_
*LOC101114495_1*	A-kinase anchor protein 17 A	-7.128	0.0244
*SCD*	Stearoyl-CoA desaturase	-4.824	1.24E-16
*LDLR*	Low density lipoprotein receptor	-4.493	8.76E-07
*RAMP2*	Receptor activity modifying protein 2	-3.766	0.022
*INSIG1*	Insulin induced gene 1	-3.730	2.43E-19
*LOC132657867*	Large ribosomal subunit protein uL16-like	2.476	0.031
*LOC132659401*	Uncharacterized	2.558	0.026
*LOC114110105*	Uncharacterized	2.618	0.041
*LOC132659352*	Uncharacterized	3.588	0.003
*CAPN13*	Calpain 13	5.244	0.004

### PPI network of microalgae oil-intake associated DEGs

**Fig. 3:**
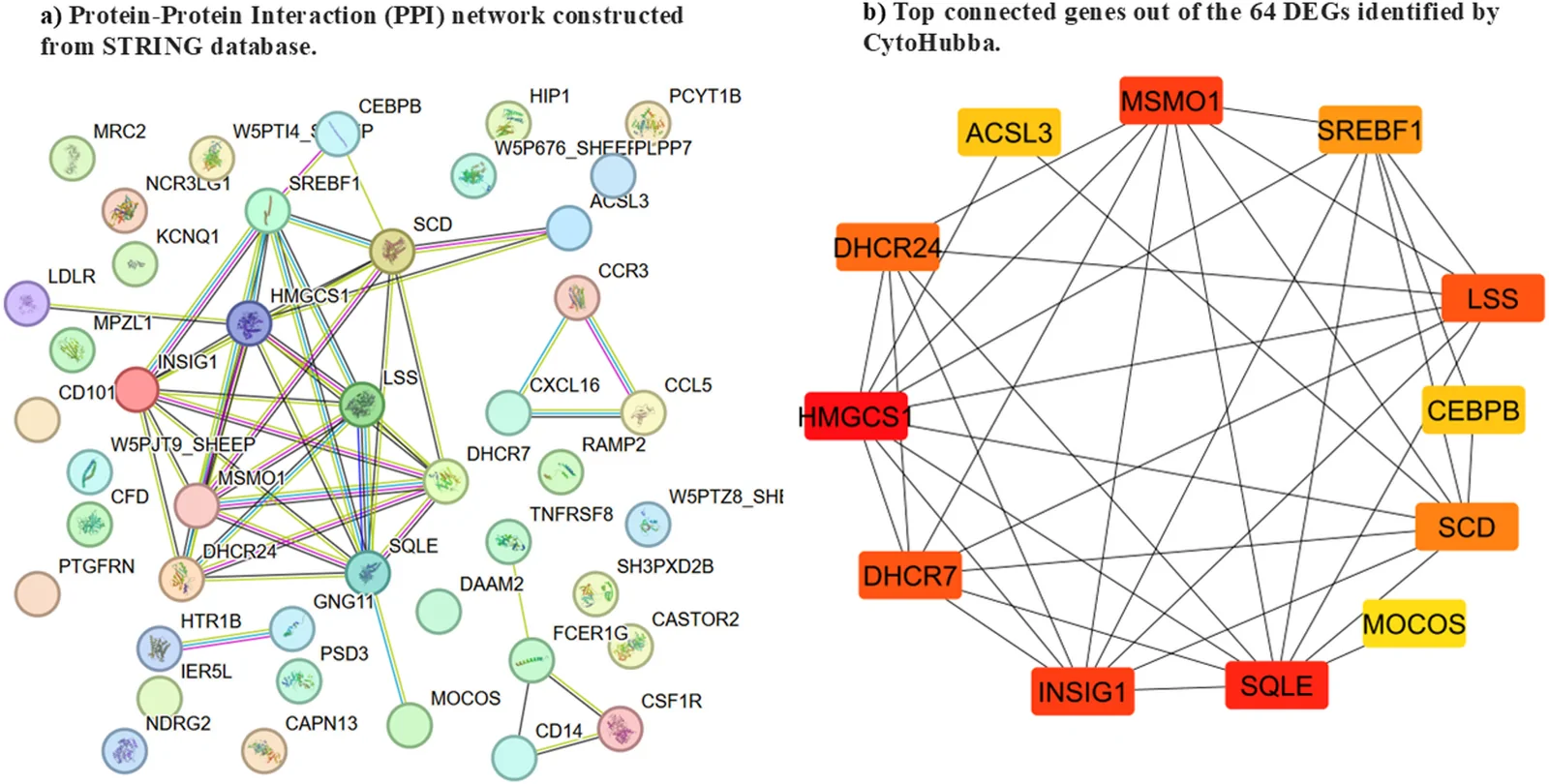
PPI Network of DEGs associated with increasing levels of microalgae oil (g/kg BW). **a** The network constructed from STRING database consisting of 44 nodes and 46 edges, highlighting significant interactions (PPI enrichment *P*: 1.0e-16). **b** Top connected genes in the network as identified by the CytoHubba plugin based on the MCC algorithm. The colour codes represent the scale of importance with red being most important gene and yellow being the least important gene

In lambs fed varying replacement levels of microalgae oil for soya oil, the PPI network identified a significantly enriched interaction landscape (PPI enrichment *P*: 1.0e-16), showing a strong connectivity (interaction score greater than 0.4) among the DEGs (Fig. 3a). A stringent interaction score cutoff of greater than 0.7 was also applied as a sensitivity analysis and is presented in Fig S1. Key downregulated genes, including 3-hydroxy-3-methylglutaryl-CoA synthase (*HMGCS1)*,* SQLE*,* MSMO1*, insulin-induced gene 1 (*INSIG1)*,* LSS*,* DHCR7*, and *DHCR24*, were central to the network (also in both medium and high-confidence interaction score cutoffs), and among the top hub genes (MCC score > 200), as identified using the CytoHubba plug-in in Cytoscape software (Fig. 3b). These genes were found to primarily interact with other genes involved in steroid biosynthesis.

### Host gene expression signatures and pathways associated with increased CH_4_ production

**Fig. 4:**
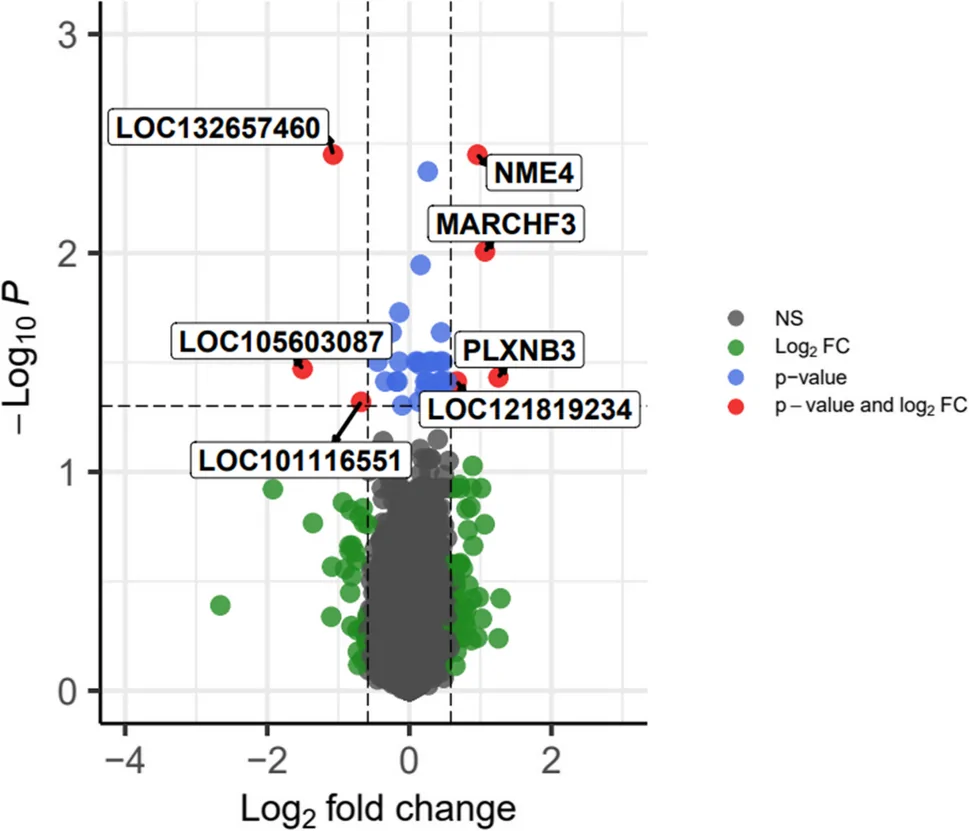
DEGs associated with lambs’ CH_4_ production (g/day). The volcano plot shows Log_2_ fold change on the x-axis and -Log_10_ *P* on the y-axis. Red colour dots represent genes that passed both *P*_*adjusted*_ and |Log_2_ fold change| cutoff. Green dots represent genes that only satisfy Log2 fold change cutoff, and blue dots represent genes that passed *P*_*adjusted*_ cutoff. Grey dots represent NS genes

From the DGE analysis with CH_4_ production (g/day) treated as a continuous phenotype, no genes passed the strict |Log2 fold change| cut off of 2. Therefore, to examine subtle transcriptional responses associated with CH_4_ production, a less-strict cut off of 0.584, corresponding to a 1.5-fold change [36] was applied resulting in seven DEGs- four upregulated (*LOC121819234*,* NME4*,* MARCHF3*, and *PLXNB3)* and three downregulated *(LOC105603087*,* LOC132657460*, and *LOC101116551* (Fig. 4; Table 3). GO enrichment analysis revealed *NME4* overrepresentation in multiple GO terms including purine nucleoside triphosphate biosynthetic process (GO:0009145), ribonucleoside triphosphate biosynthetic process (GO:0009201), and pyrimidine-containing compound metabolic process (GO:0072527) (see Additional file 5). Furthermore, upregulation of *NME4* gene significantly enriched five KEGG pathways: “pyrimidine metabolism”, “drug metabolism”, “nucleotide metabolism”, “purine metabolism”, and “biosynthesis of cofactors”, while *PLXNB3* upregulation enriched the “axon guidance pathway” (see Additional file 5). The remaining five genes showed no significant pathway enrichment due to the uncharacterized or incompletely annotated functions rather than the absence of biological relevance.

**Table 3:** DEGs associated with CH_4_ production identified from DGE analysis. The significant threshold was determined as *P*_*adjusted*_ < 0.05, and absolute |Log_2_ fold change| = 0.584 (1.5-fold change) where *P*_*adjusted*_ was calculated by adjusting *P* from Wald test using BH method. CH_4_ production was treated as a continuous phenotype. Hence Log_2_ fold change here corresponds to fold change per unit increase in CH_4_ production

Gene ID	Gene name	Gene Function	Log_2_ fold change	*P*_adjusted_
*LOC132657460*	Uncharacterized		-1.072	0.003
*NME4*	NME/NM23 nucleoside diphosphate kinase 4	cellular differentiation, proliferation, apoptosis [37], and catabolism of short-chain fatty acids (SCFA) [38]	0.957	0.003
*MARCHF3*	Membrane associated ring-CH-type finger 3	regulates immune responses [39]	1.065	0.009
*LOC105603087*	Myeloid-associated differentiation marker-like		-1.502	0.033
*PLXNB3*	Plexin B3	semaphorin signalling pathway, axonal guidance [40], regulating morphology and motility of various cell types [41]	1.253	0.036
*LOC121819234*	Uncharacterized		0.669	0.038
*LOC101116551*	GTPase IMAP family member 1-like		-0.679	0.047

Linear regression analysis confirmed five out of seven DEGs with significant CH_4_ production-associated effects (*P*_*adjusted*_ < 0.05) (Table 4, Fig S2). Among these genes, *NME4* displayed the strongest significant effects (R^2^ of 0.58, β = 2.38). Moderate to strong significant effects were also observed for *LOC121819234* and *LOC101116551*, with a negative gene expression-CH_4_ relationship for the latter gene. Comparatively weak effects were observed for *LOC132657460* and *PLXNB3*, however these effects were still statistically significant. *MARCHF3* and *LOC105603087* gene expression displayed non-significant effects with CH_4_ production.

**Table 4:** Association between gene expression and CH_4_ production for the seven DEGs. The table shows regression coefficients (β), coefficient of determination (R²), and BH-corrected *P* (*P*_*adjuste****d***_) for the linear models which evaluated the association between CH_4_ production and normalized expression of the seven CH_4_‑associated genes identified from DESeq2

Gene	Slope (β)	Coefficient of determination (*R*^2^)	*P*_adjusted_
*LOC132657460*	-2.58	0.38	0.006
*NME4*	2.38	0.58	0.0006
*MARCHF3*	7.19	0.14	0.09
*LOC105603087*	-34.12	0.17	0.08
*PLXNB3*	1.40	0.29	0.02
*LOC121819234*	1.05	0.51	0.001
*LOC101116551*	-2.16	0.47	0.001

### Functional clustering of DEGs using GO semantic similarity

Distinct gene semantic similarity patterns were observed for microalgae oil intake-associated DEGs across the three GO categories: CC, MF and BP. The heatmap generated displayed functional relationship between genes with red colour representing GO annotations that are closely related (high semantic similarity), orange or light orange with moderate similarities and blue with low similarities (Fig S3). Several gene pairs with moderate similarities were observed for CC category (Fig S3a) while high similarities between gene-pairs were found for the MF category (Fig S3b). The BP ontology displayed greatest heterogeneity with most of the genes showing lower semantic similarities (Fig S3c). Importantly, seven hub genes identified from PPI network (*DHCR7*,* DHCR24*,* HMGCS1*,* INSIG1*,* LSS*,* MSMO1*, and *SQLE*) displayed moderate to high semantic similarities, while four hub genes- *ACSL3*,* CEBPB*, *SREBF1*, and *SCD* exhibited low similarity scores (Fig S3).

Three (*NME4*,* MARCHF3*,* PLXNB3*) out of seven CH_4_-associated DEGs were successfully mapped to their corresponding human orthologs. Heatmap showing semantic similarity scores between these three genes using CC, MF and BP ontologies are represented in Fig S4. *PLXNB3* displayed low similarity with both *NME4* and *MARCHF3*, while *NME4* and *MARCHF3* exhibited moderate similarity for the CC category (Fig S4a). Almost similar patterns were observed for MF and BP category with low similarities between all three genes (Fig S4b, c). Incorporating a relaxed threshold of genes (*P*_adjusted_ < 0.05 and |Log_2_ fold change| > 0) resulted in 33 genes with all three ontologies displaying substantial similarities particularly for MF ontology (Fig S5). Furthermore, we could not find any gene cluster semantic similarities between the microalgae oil intake-associated DEGs and CH_4_-associated DEGs.

## Discussion

The implications of CH_4_ production from ruminants raise serious concerns about both the environmental sustainability and the energy efficiency of food production practices associated with sheep farming. To address this issue, while genetic selection of animals with low CH_4_ production would be a cost-efficient, and permanent strategy across generations [42, 43], dietary interventions remain an important alternative approach. In this study, feed supplementation with varying levels of microalgae oil over a nine-week period did not reveal any statistically significant differences in CH_4_ production, suggesting that under the tested conditions, group-level inclusion to diet was not sufficient to alter CH_4_ production.

No significant changes in bodyweight, total DMI or ADG in response to dietary microalgae oil were observed suggesting no changes in feed intake patterns or animal growth performance. These findings are consistent with previous studies which observed no significant growth performance effects from microalgae oil consumption in swine, lambs, and calves [44–46]. These observations are particularly relevant as dietary interventions using microalgae oil that aim to reduce CH_4_ production may not compromise or negatively affect other production traits including intake or overall growth. Therefore, any changes observed in CH_4_ production levels in the animals in this study are unlikely to be due to differences in microalgae oil or differences in feed intake.

To uncover the gene expression profiles associated with varying levels of microalgae oil, DGE analysis was conducted using actual microalgae oil intake treated as continuous exposure rather than group-level inclusion. Treating microalgae oil intake as continuous variable improves statistical power and helps in identifying genes with even subtle expression changes that might get overlooked through a group-level inclusion analysis. Dichotomizing actual microalgae oil intake levels into discrete groups may result in significant information loss as a result of collapsing individual intake variations into different groups. However, preserving the complete numerical intake values of microalgae oil (as they represent real biological measurement scale) enables continuous statistical models to utilise even minute differences between intake levels, increasing sensitivity and precision [47].

Six key genes out of total 64 DEGs- *HMGCS1*, *INSIG1*, sterol regulatory element binding transcription factor 1 (*SREBF1*), *DHCR24*, *DHCR7*, and *LSS-* were consistently downregulated across several enriched biological terms (sterol metabolism, steroid biosynthesis, and lipid biosynthesis) and were also among the hub genes in the PPI network pointing to a collective transcriptional response towards varying levels of dietary microalgae oil. Although the exact drivers of this expression pattern cannot be confirmed, downregulation of these six genes are consistent with known regulatory effects of long-chain n-3 polyunsaturated fatty acids (LC-n3 PUFA), particularly eicosapentaenoic acid (EPA) and docosahexaenoic acid (DHA), which are abundant in microalgae oil [48]. Similar findings were reported in a study where male hamsters fed with diets enriched in PUFA displayed approximately 60% in vivo sterol synthesis reduction [49]. These six hub genes were also reported to have critical roles in cholesterol and sterol biosynthetic pathways, with *HMGCS1* catalysing the conversion of acetyl-CoA and acetoacetyl-CoA into HMG-CoA, a compound that plays a pivotal role in cholesterol synthesis [50]. *INSIG1* is a lipogenic gene activated by *SREBF1* [51] that plays a role in regulating cholesterol synthetic genes [52]. *DHCR24*, *DHCR7*, and *LSS* are crucial genes at different stages of the steroid biosynthetic pathway: *LSS* catalyses the conversion of (S)-2,3-oxidosqualene to lanosterol [53], and *DHCR7* and *DHCR24* reduce the delta-7 and delta-24 double bonds in sterol intermediates [54, 55]. Therefore, a combined downregulation of these genes’ points to an altered biosynthesis of cholesterol in lambs, likely as a consequence of increased microalgae oil within ingested feed. Such downregulation may suggest either a protective or a compensatory response, with genes involved in cholesterol biosynthesis displaying an initial increase in expression within rumen epithelial cells of dairy cattle during the first week of a high-grain diet, and subsequently downregulated by week 3 [56]. Elevated cellular levels of cholesterol can lead to inflammation, oxidative stress, altered cell growth, and changes in membrane function [56]. Therefore, downregulation of cholesterol biosynthetic genes observed in this study suggest that cholesterol biosynthesis is reduced with increasing microalgae oil consumption. RNA-Seq was carried out on blood samples collected post-feeding, without paired pre-feeding transcriptomic profiles as a baseline reference. Hence, although the enrichment of lipid metabolism, cholesterol biosynthesis and steroid biosynthetic pathways supports a biologically plausible association with varying microalgae oil, this analysis cannot fully distinguish microalgae oil effects from pre-existing inter-individual variations. These genes should therefore be interpreted as post-feeding blood-derived transcriptional signatures associated with microalgae oil intake. Nevertheless, observed downregulation may reflect a compensatory mechanism to prevent the overproduction of sterols, which, at elevated concentrations may otherwise potentially induce inflammation, oxidative stress, cell proliferation, and membrane permeability changes, leading to tissue damage [50].

CH_4_ production was observed to vary inter-individually across the study lambs with a negative correlation between CH_4_ production and actual intake of microalgae oil observed. We used soya oil to balance the microalgae oil, thus minimizing the effect of oil itself on CH_4_ production. Evidence indicates that dietary supplementation with any type of oil can influence rumen fermentation and reduce CH_4_ emissions [57, 58]. Therefore, the present design could minimize the effect of oil itself but explore the effect of anti-methanogenesis function of microalgae oil. It is often assumed that CH_4_ production varies with feed consumption or growth rate; but lack of significant correlation between CH_4_ production and DMI or final body weight of lambs in this study suggests that CH_4_ production may be influenced by other factors (biological or genetic factors) beyond total feed intake or growth such as host metabolic factors, digestive efficiency, or rumen fermentation patterns. As such, CH_4_ production, when analyzed as continuous phenotype, enabled the identification of host transcriptional responses associated with inter-individual CH_4_ differences, with seven significantly associated genes. Notably, none of these genes overlapped with the set of genes (*n* = 64 DEGs) identified in response to microalgae oil, suggesting different regulatory or functional roles for these two sets of genes, potentially pointing to non-overlapping biological mechanisms driving inherent CH_4_ production and dietary responses to microalgae oil. This was further confirmed through the gene cluster semantic similarity analysis where we could not find any semantic similarities between the two gene clusters suggesting no functional overlap. Genes including *NME4*, *LOC121819234*,* MARCHF3*, and *PLXNB3* were found to be upregulated in lambs with higher CH_4_ production. *NME4* encodes mitochondrial nucleoside di-phosphate kinase (NDPK) and its upregulation suggests a possible connection between mitochondrial functioning and energy metabolism which are associated with CH_4_ production, as reduced efficiency of energy use in animals may result in greater availability of hydrogen for methanogenesis in the rumen. *LOC121819234* is an uncharacterized gene, whereas *MARCHF3*, normally expressed in immune cells such as B cells, and T cells, reflects host immune responses towards microbial activities in the rumen [39]. *PLXNB3* regulates the morphology and motility of several immune cells [41]. Few studies have reported *PLXNB3* gene and its associations with growth [59], stature [60], and carcass quality [61], however no studies were found reporting its association with CH_4_ production. Whilst the role of *PLXNB3* in CH_4_ production is unknown, the association identified in the current study may suggest a broad regulatory role of the *PLXNB3* gene in connecting host physiology to CH_4_ production which is correlated to efficient fermentation. Furthermore, immune system associated roles of *MARCHF3* and *PLXNB3* suggest that upregulation of these genes may disturb the immune system functions in lambs with inherently increased CH_4_ production. Uncharacterized *LOC132657460*,* LOC105603087*, and *LOC101116551* were the three downregulated DEGs associated with increasing CH_4_ production. *LOC105603087*, annotated as *MYADM*-like gene is expressed in hematopoietic cells with putative links to cell migration, myeloid differentiation and lamb weight traits [62, 63]. Knockdown of *MYADM* has been associated with an inflammatory-like phenotype and altered barrier function [64]. Similarly, *LOC101116551* belongs to GIMAP1 proteins with roles in regulating lymphocyte survival and maintaining homeostasis. Altered GIMAP1 expressions were associated with inflammatory and autoimmune diseases [65], particularly, its downregulation was observed in cattle with Johne’s disease [66] confirming an immune resilience role. However, it should be noted that any functional interpretation of uncharacterized genes (i.e. *LOC132657460*, *LOC105603087*,* LOC101116551*) should remain cautious due to limited characterization in sheep. Nevertheless, based on their predicted functions, the observed downregulation of these two genes may suggest a weak functioning of myeloid and lymphocytes in lambs that emit more CH_4_, but does not establish a causal role in CH_4_ production. Therefore, further studies on functionally annotating such uncharacterized genes are critical in sheep research.

Importantly, *MARCHF3* and *LOC105603087*, among the seven CH_4_-associated DEGs did not retain significance in the linear regression analyses. This difference possibly reflects the different statistical methods of the two analyses, as DGE analysis find genes with significant changes in expression across samples whereas linear regression analyses investigate the association strength between the continuous CH_4_ measurement and gene expression. Despite these differences, consistent identification of five CH_4_-associated genes from both approaches provides strong evidence for the involvement of these genes in CH_4_‑related physiological processes. While these candidate genes contribute new insight into potential regulatory mechanisms influencing CH_4_ production, RT-qPCR validation as well as replication in an independent or larger population were beyond the scope of the present work. These steps will be incorporated into future studies to further support and refine the gene-CH_4_ associations identified here.

Blood transcriptomics in this study should be interpreted as a proxy for systemic gene expression responses in the host, rather than as a direct representation of transcriptional activities in the rumen. However, the current study provides host-associated molecular information complementary to approaches focused on rumen sampling methods, although integrative analyses utilizing blood, rumen tissues, microbiome, and metabolomics will be needed to fully resolve tissue-specific mechanisms underlying CH₄ variation.

## Conclusions

The present study has reported alterations in the gene expression profiles of lambs supplemented with varying levels of microalgae oil, while also identifying host genes linked to CH_4_ production. Different levels of microalgae oil (group-allocation) replacement were shown to not significantly influence CH_4_ production or growth performance, but to have a positive impact on the metabolic functioning of lambs by altering blood gene expression leading to downregulation of genes (*HMGCS1*,* SQLE*,* MSMO1*,* INSIG1*,* LSS*,* DHCR7*, *DHCR24*) associated with steroid biosynthesis and other lipid metabolic pathways. In addition, various DEGs (including *NME4*,* MARCHF3*, and *PLXNB3)* associated with CH_4_ production were identified that participate in key biological processes including immune regulation and cellular differentiation. Whilst exact roles in CH_4_ production remains to be elucidated, involvement in immune-related activities highlights the need for further investigations to examine how external factors such as infection status or overall health might influence these genes and their connection to CH_4_ production. Findings also signify that CH_4_ production is governed by inter-individual differences in part and underscores the importance of incorporating host-level mechanisms alongside dietary strategies for CH_4_ mitigation programmes, thereby promoting more sustainable and environmentally responsible agricultural practices.

## Supplementary Information

Supplementary Material 1.

## Data Availability

The datasets generated and analysed during the current study are deposited in the NCBI sequence read archive (SRA) repository, BioProject ID: PRJNA1451055, which can be accessed at -https://eur02.safelinks.protection.outlook.com/?url=https%3A%2F%2Feu-west-1.protection.sophos.com%2F%3Fd%3Dnih.gov%26u%3DaHR0cDovL3d3dy5uY2JpLm5sbS5uaWguZ292L2Jpb3Byb2plY3QvMTQ1MTA1NQ%3D%3D%26i%3DNjU1NTMyNDY4MjQyNDMyZTkzMTQwMTIw%26t%3DR2RTY09UNzZOVjgzZkpoazNaQlBMRVVPWjMxZ2lTTjQ3aS9kNGh2OTBCcz0%3D%26h%3D55b6147562614c178ba4051f841b8b41%26s%3DAVNPUEhUT0NFTkNSWVBUSVaGtG3OaKxlTcq0fhGjKKcMkmp_laJ8K5UdFPgZa4vZPQ&data=05%7C02%7Cschackokaitholil01%40qub.ac.uk%7Ca6d62488b2ef4efb57d008de962b3e51%7Ceaab77eab4a549e3a1e8d6dd23a1f286%7C0%7C0%7C639113309646423665%7CUnknown%7CTWFpbGZsb3d8eyJFbXB0eU1hcGkiOnRydWUsIlYiOiIwLjAuMDAwMCIsIlAiOiJXaW4zMiIsIkFOIjoiTWFpbCIsIldUIjoyfQ%3D%3D%7C0%7C%7C%7C&sdata=qMbH0rAt20%2BuuIJvx2wVfhjvAf3%2B92pOC8kjor5hcPI%3D&reserved=0.

## Abbreviations

DEG: Differentially expressed gene
DGE: Differential gene expression
*DHCR7*: 7-dehydrocholesterol reductase
*DHCR24*: 24-dehydrocholesterol reductase
DMI: Dry matter intake
GO: Gene ontology
*HMGCS1*: 3-hydroxy-3-methylglutaryl-CoA synthase
*INSIG1*: Insulin-induced gene 1
*KEGG*: Kyoto encyclopaedia of genes and genomes
*LSS*: Lanosterol synthase
*LOC105603087*: Myeloid-associated differentiation marker-like
*LOC101116551*: GTPase IMAP family member 1-like
*MARCHF3*: Membrane associated ring-CH-type finger 3
*MSMO1*: Methyl sterol monooxygenase 1
*NME4*: NME/NM23 nucleoside diphosphate kinase 4
ORA: Overrepresentation analysis
*PLXNB3*: Plexin B3
*SQLE*: Squalene epoxidase

## Footnotes

1
https://www.ncbi.nlm.nih.gov/datasets/genome/GCF_016772045.2/.

## References

[1] Kim M, Masaki T, Ikuta K, Iwamoto E, Nishihara K, Hirai M, et al. Physiological responses and adaptations to high methane production in Japanese Black cattle. Sci Rep. 2022;12(1):11154. 35778422 10.1038/s41598-022-15146-1 PMC9249741

[2] Van Nevel C, Demeyer D. Control of rumen methanogenesis. Environ Monit Assess. 1996;42:73–97. 24193494 10.1007/BF00394043

[3] Caro D, Kebreab E, Mitloehner FM. Mitigation of enteric methane emissions from global livestock systems through nutrition strategies. Clim Change. 2016;137:467–80.

[4] Mohammed N, Lila Z, Ajisaka N, Hara K, Mikuni K, Hara K, et al. Inhibition of ruminal microbial methane production by β-cyclodextrin iodopropane, malate and their combination in vitro. J Anim Physiol Anim Nutr. 2004;88(5–6):188–95. 10.1111/j.1439-0396.2004.00456.x 15189423

[5] Benchaar C, Calsamiglia S, Chaves AV, Fraser G, Colombatto D, McAllister TA, et al. A review of plant-derived essential oils in ruminant nutrition and production. Anim Feed Sci Technol. 2008;145(1–4):209–28.

[6] Vítor A, Godinho M, Francisco A, Silva J, Almeida J, Fialho L, et al. Nannochloropsis oceanica microalga feeding increases long-chain omega-3 polyunsaturated fatty acids in lamb meat. Meat Sci. 2023;197:109053. 36493555 10.1016/j.meatsci.2022.109053

[7] Fievez V, Dohme F, Danneels M, Raes K, Demeyer D. Fish oils as potent rumen methane inhibitors and associated effects on rumen fermentation in vitro and in vivo. Anim Feed Sci Technol. 2003;104(1–4):41–58.

[8] Maia MR, Fonseca AJ, Oliveira HM, Mendonça C, Cabrita AR. The potential role of seaweeds in the natural manipulation of rumen fermentation and methane production. Sci Rep. 2016;6(1):32321. 27572486 10.1038/srep32321 PMC5004155

[9] Mavrommatis A, Sotirakoglou K, Skliros D, Flemetakis E, Tsiplakou E. Dose and time response of dietary supplementation with Schizochytrium sp. on the abundances of several microorganisms in the rumen liquid of dairy goats. Livest Sci. 2021;247:104489.

[10] Altomonte I, Salari F, Licitra R, Martini M. Use of microalgae in ruminant nutrition and implications on milk quality–A review. Livest Sci. 2018;214:25–35.

[11] Pinares-Patiño C, Hickey S, Young E, Dodds K, MacLean S, Molano G, et al. Heritability estimates of methane emissions from sheep. animal. 2013;7:316–21. 23739473 10.1017/S1751731113000864 PMC3691003

[12] Shi W, Moon CD, Leahy SC, Kang D, Froula J, Kittelmann S, et al. Methane yield phenotypes linked to differential gene expression in the sheep rumen microbiome. Genome Res. 2014;24(9):1517–25. 24907284 10.1101/gr.168245.113 PMC4158751

[13] Xiang R, McNally J, Bond J, Tucker D, Cameron M, Donaldson AJ, et al. Across-experiment transcriptomics of sheep rumen identifies expression of lipid/oxo-acid metabolism and muscle cell junction genes associated with variation in methane-related phenotypes. Front Genet. 2018;9:330. 30177952 10.3389/fgene.2018.00330 PMC6109778

[14] Razban V, Carballo OC, Morrison S, Shirali M. RNA-Seq Analysis of Ruminal Methane Emissions in Beef-on-Dairy Cattle: Evidence for Immune, Nervous, and Endocrine Pathway Involvement. Animals. 2026;16(4):589. 41751050 10.3390/ani16040589 PMC12937309

[15] Mrutu RI, Abdussamad AM, Umar KM, Abdulhamid A, Farny NG. Mitigating methane emissions and promoting acetogenesis in ruminant livestock. Front Anim Sci. 2025;6:1489212.

[16] Krištolaitytė J, Džermeikaitė K, Rutkauskas A, Šertvytytė G, Lembovičiūtė G, Arlauskaitė S, et al. Impact of Blood Metabolic Profile and Ingestive Behaviours Registered with Noseband Sensor on Methane Emission During Transition Period in Dairy Cows. Life. 2025;15(5):760. 40430188 10.3390/life15050760 PMC12113304

[17] Lee H, Kim M, Masaki T, Ikuta K, Iwamoto E, Oikawa K, et al. Exploring the link between ruminal methane production and physiological changes in Japanese Black cattle during fattening. Scientific Reports. 2026;16(5915). 10.1038/s41598-026-36644-6 PMC12895014 41565990

[18] Hagey JV, Laabs M, Maga EA, DePeters EJ. Rumen sampling methods bias bacterial communities observed. PLoS ONE. 2022;17(5):e0258176. 35511785 10.1371/journal.pone.0258176 PMC9070869

[19] Fresco S, Boichard D, Fritz S, Lefebvre R, Barbey S, Gaborit M, et al. Comparison of methane production, intensity, and yield throughout lactation in Holstein cows. J Dairy Sci. 2023;106(6):4147–57. 37105882 10.3168/jds.2022-22855

[20] Cristobal-Carballo O, Santos FG, Morrison S, Huws S, Brans J, Aubry A, et al. Effect of different levels of microalgae supplementation on methane emissions, ruminal fermentation, dietary digestibility and nitrogen retention in finishing lambs. Animal-science proceedings. 2021;12(1):63.

[21] Sharon Huws OC-C, Godoy-Santos F, Morrison S, Aubry A. Johanna Brans, Eva Lewis, Tianhai Yan, editor Microalgae as feed. British Society of Animal Science; 2023: Elsevier; 2023.

[22] Andrews S. FastQC: a quality control tool for high throughput sequence data. Cambridge, United Kingdom; 2010; http://www.bioinformatics.babraham.ac.uk/projects/fastqc/ ; (10 January 2025, date last accessed).

[23] Bolger AM, Lohse M, Usadel B. Trimmomatic: a flexible trimmer for Illumina sequence data. Bioinformatics. 2014;30(15):2114–20. 24695404 10.1093/bioinformatics/btu170 PMC4103590

[24] Kim D, Paggi JM, Park C, Bennett C, Salzberg SL. Graph-based genome alignment and genotyping with HISAT2 and HISAT-genotype. Nat Biotechnol. 2019;37(8):907–15. 31375807 10.1038/s41587-019-0201-4 PMC7605509

[25] Liao Y, Smyth GK, Shi W. featureCounts: an efficient general purpose program for assigning sequence reads to genomic features. Bioinformatics. 2014;30(7):923–30. 24227677 10.1093/bioinformatics/btt656

[26] Love MI, Huber W, Anders S. Moderated estimation of fold change and dispersion for RNA-seq data with DESeq2. Genome Biol. 2014;15:1–21. 10.1186/s13059-014-0550-8 PMC4302049 25516281

[27] Sha Y, Phan JH, Wang MD, editors. Effect of low-expression gene filtering on detection of differentially expressed genes in RNA-seq data. 2015 37th Annual International Conference of the IEEE Engineering in Medicine and Biology Society (EMBC); 2015:IEEE. 10.1109/EMBC.2015.7319872 PMC4983442 26737772

[28] Ihaka R, Gentleman R. R: a language for data analysis and graphics. J Comput graphical Stat. 1996;5(3):299–314.

[29] Gao L, Zhang Y, Zhang Y, Peng W, Zhang Z, Liu Y, et al. Transcriptome sequencing unveils a novel mechanism underlying breed distinctions between thin-and fat-tailed sheep. Genes. 2026;17(2):162. 41751546 10.3390/genes17020162 PMC12940624

[30] Yu G, Wang L-G, Han Y, He Q-Y. clusterProfiler: an R package for comparing biological themes among gene clusters. OMICS. 2012;16(5):284–7. 22455463 10.1089/omi.2011.0118 PMC3339379

[31] Morgan M, Carlson M, Tenenbaum D, Arora S. Package ‘AnnotationHub’. Package ‘AnnotationHub’. 2019.

[32] Szklarczyk D, Kirsch R, Koutrouli M, Nastou K, Mehryary F, Hachilif R, et al. The STRING database in 2023: protein–protein association networks and functional enrichment analyses for any sequenced genome of interest. Nucleic Acids Res. 2023;51(D1):D638–46. 36370105 10.1093/nar/gkac1000 PMC9825434

[33] Shannon P, Markiel A, Ozier O, Baliga NS, Wang JT, Ramage D, et al. Cytoscape: a software environment for integrated models of biomolecular interaction networks. Genome Res. 2003;13(11):2498–504. 14597658 10.1101/gr.1239303 PMC403769

[34] Yu G, Li F, Qin Y, Bo X, Wu Y, Wang S. GOSemSim: an R package for measuring semantic similarity among GO terms and gene products. Bioinformatics. 2010;26(7):976–8. 20179076 10.1093/bioinformatics/btq064

[35] Kolde R, Kolde MR. Package ‘pheatmap’. R package. 2015;1(7):790.

[36] Wang G, Tian L, Zhang S, He Z, Zhao F, Chang M, et al. Deciphering the Regulatory Network of Tail Fat Deposition in Large-and Small-Tailed Han Sheep Through Transcriptome and MicroRNAome Profiling. Biology. 2026;15(2):179. 41594914 10.3390/biology15020179 PMC12838090

[37] Boissan M, Schlattner U, Lacombe M-L. The NDPK/NME superfamily: state of the art. Elsevier; 2018. pp. 164–74. 10.1038/labinvest.2017.137 29451272

[38] Tokarska-Schlattner M, Boissan M, Munier A, Borot C, Mailleau C, Speer O, et al. The nucleoside diphosphate kinase D (NM23-H4) binds the inner mitochondrial membrane with high affinity to cardiolipin and couples nucleotide transfer with respiration. J Biol Chem. 2008;283(38):26198–207. 18635542 10.1074/jbc.M803132200 PMC3258864

[39] Kim CH, Park J, Kim M. Gut microbiota-derived short-chain fatty acids, T cells, and inflammation. Immune Netw. 2014;14(6):277. 25550694 10.4110/in.2014.14.6.277 PMC4275385

[40] Liu Z-Z, Liu L-Y, Zhu L-Y, Zhu J, Luo J-Y, Wang Y-F, et al. Plexin B3 guides axons to cross the midline in vivo. Front Cell Neurosci. 2024;18:1292969. 38628398 10.3389/fncel.2024.1292969 PMC11018898

[41] Alto LT, Terman JR. Semaphorins and their Signaling Mechanisms. Methods Mol Biol. 2017;1493:1–25. 27787839 10.1007/978-1-4939-6448-2_1 PMC5538787

[42] Jalil Sarghale A, Moradi Shahrebabak M, Moradi Shahrebabak H, Nejati Javaremi A, Saatchi M, Khansefid M, et al. Genome-wide association studies for methane emission and ruminal volatile fatty acids using Holstein cattle sequence data. BMC Genet. 2020;21:1–14. 33228565 10.1186/s12863-020-00953-0 PMC7684878

[43] de Haas Y, Veerkamp R, de Jong G, Aldridge M. Selective breeding as a mitigation tool for methane emissions from dairy cattle. Animal. 2021;15:100294. 34246599 10.1016/j.animal.2021.100294

[44] Abril R, Garrett J, Zeller SG, Sander WJ, Mast RW. Safety assessment of DHA-rich microalgae from Schizochytrium sp. Part V: target animal safety/toxicity study in growing swine. Regul Toxicol Pharmacol. 2003;37(1):73–82. 12662911 10.1016/s0273-2300(02)00030-2

[45] Meale S, Chaves A, He M, McAllister T. Dose–response of supplementing marine algae (Schizochytrium spp.) on production performance, fatty acid profiles, and wool parameters of growing lambs. J Anim Sci. 2014;92(5):2202–13. 24668954 10.2527/jas.2013-7024

[46] Flaga J, Korytkowski L, Gorka P, Kowalski ZM. The effect of docosahexaenoic acid-rich algae supplementation in milk replacer on performance and selected immune system functions in calves. J Dairy Sci. 2019;102(10):8862–73. 31421880 10.3168/jds.2018-16189

[47] Lazic SE. Why we should use simpler models if the data allow this: relevance for ANOVA designs in experimental biology. BMC Physiol. 2008;8(1):16. 18644134 10.1186/1472-6793-8-16 PMC2496911

[48] Khavari F, Saidijam M, Taheri M, Nouri F. Microalgae: therapeutic potentials and applications. Mol Biol Rep. 2021;48(5):4757–65. 34028654 10.1007/s11033-021-06422-w PMC8142882

[49] Schmid KE, Woollett LA. Differential effects of polyunsaturated fatty acids on sterol synthesis rates in adult and fetal tissues of the hamster: consequence of altered sterol balance. Am J Physiology-Gastrointestinal Liver Physiol. 2003;285(5):G796–803. 10.1152/ajpgi.00226.2003 14561586

[50] Cotter DG, Ercal B, Huang X, Leid JM, d’Avignon DA, Graham MJ, et al. Ketogenesis prevents diet-induced fatty liver injury and hyperglycemia. J Clin Invest. 2014;124(12):5175–90. 25347470 10.1172/JCI76388 PMC4348980

[51] Nafikov RA, Schoonmaker JP, Korn KT, Noack K, Garrick DJ, Koehler KJ, et al. Sterol regulatory element binding transcription factor 1 (SREBF1) polymorphism and milk fatty acid composition. J Dairy Sci. 2013;96(4):2605–16. 23403193 10.3168/jds.2012-6075

[52] Horton JD, Shah NA, Warrington JA, Anderson NN, Park SW, Brown MS, et al. Combined analysis of oligonucleotide microarray data from transgenic and knockout mice identifies direct SREBP target genes. Proc Natl Acad Sci U S A. 2003;100(21):12027–32. 14512514 10.1073/pnas.1534923100 PMC218707

[53] Mori M, Li G, Abe I, Nakayama J, Guo Z, Sawashita J, et al. Lanosterol synthase mutations cause cholesterol deficiency–associated cataracts in the Shumiya cataract rat. J Clin Investig. 2006;116(2):395–404. 16440058 10.1172/JCI20797 PMC1350995

[54] Prabhu AV, Luu W, Sharpe LJ, Brown AJ. Phosphorylation regulates activity of 7-dehydrocholesterol reductase (DHCR7), a terminal enzyme of cholesterol synthesis. J Steroid Biochem Mol Biol. 2017;165(Pt B):363–8. 27520299 10.1016/j.jsbmb.2016.08.003

[55] Zheng HS, Kang Y, Lyu Q, Junghans K, Cleary C, Reid O, et al. DHCR24, a key enzyme of cholesterol synthesis, serves as a marker gene of the mouse adrenal gland inner cortex. Int J Mol Sci. 2023;24(2):933. 36674444 10.3390/ijms24020933 PMC9867314

[56] Steele MA, Vandervoort G, AlZahal O, Hook SE, Matthews JC, McBride BW. Rumen epithelial adaptation to high-grain diets involves the coordinated regulation of genes involved in cholesterol homeostasis. Physiol Genomics. 2011;43(6):308–16. 21245418 10.1152/physiolgenomics.00117.2010

[57] Bayat AR, Tapio I, Vilkki J, Shingfield K, Leskinen H. Plant oil supplements reduce methane emissions and improve milk fatty acid composition in dairy cows fed grass silage-based diets without affecting milk yield. J Dairy Sci. 2018;101(2):1136–51. 29224879 10.3168/jds.2017-13545

[58] Nasir M, Rodríguez-Prado M, Simoni M, Martín-Orúe SM, Pérez JF, Calsamiglia S. Optimizing Essential Oil Mixtures: Synergistic Effects on Cattle Rumen Fermentation and Methane Emission. Animals. 2025;15(14):2105. 40723567 10.3390/ani15142105 PMC12291954

[59] Chen F, Wu P, Shen M, He M, Chen L, Qiu C, et al. Transcriptome analysis of differentially expressed genes related to the growth and development of the Jinghai yellow chicken. Genes. 2019;10(7):539. 31319533 10.3390/genes10070539 PMC6678745

[60] Sanchez M-P, Escouflaire C, Baur A, Bottin F, Hozé C, Boussaha M, et al. X-linked genes influence various complex traits in dairy cattle. BMC Genomics. 2023;24(1):338. 37337145 10.1186/s12864-023-09438-7 PMC10278306

[61] Jové-Juncà T, Crespo-Piazuelo D, González-Rodríguez O, Pascual M, Hernández-Banqué C, Reixach J, et al. Genomic architecture of carcass and pork traits and their association with immune capacity. Animal. 2024;18(1):101043. 38113634 10.1016/j.animal.2023.101043

[62] Gonzalez MV, Mousel MR, Herndon DR, Jiang Y, Dalrymple BP, Reynolds JO, et al. A divergent Artiodactyl MYADM-like repeat is associated with erythrocyte traits and weight of lamb weaned in domestic sheep. PLoS ONE. 2013;8(8):e74700. 24023702 10.1371/journal.pone.0074700 PMC3758307

[63] Salgado Pardo JI, Delgado Bermejo JV, González Ariza A, León Jurado JM, Marín Navas C, Iglesias Pastrana C, et al. Candidate genes and their expressions involved in the regulation of milk and meat production and quality in goats (Capra hircus). Animals. 2022;12(8):988. 35454235 10.3390/ani12080988 PMC9026325

[64] Aranda JF, Reglero-Real N, Marcos-Ramiro B, Ruiz-Sáenz A, Fernández-Martín L, Bernabé-Rubio M, et al. MYADM controls endothelial barrier function through ERM-dependent regulation of ICAM-1 expression. Mol Biol Cell. 2013;24(4):483–94. 23264465 10.1091/mbc.E11-11-0914 PMC3571871

[65] Datta P, Webb LM, Avdo I, Pascall J, Butcher GW. Survival of mature T cells in the periphery is intrinsically dependent on GIMAP1 in mice. Eur J Immunol. 2017;47(1):84–93. 27792288 10.1002/eji.201646599 PMC5244661

[66] Thirunavukkarasu S, Plain KM, de Silva K, Begg D, Whittington RJ, Purdie AC. Expression of genes associated with cholesterol and lipid metabolism identified as a novel pathway in the early pathogenesis of Mycobacterium avium subspecies paratuberculosis-infection in cattle. Vet Immunol Immunopathol. 2014;160(3–4):147–57. 24930699 10.1016/j.vetimm.2014.04.002

