# Supplementary material for: Blood-derived gene expression profiles associated with dietary microalgae oil intake and methane emission variation in lambs
Source: BMC Genomics. 2026 Jun 23;27:737. doi: 10.1186/s12864-026-13110-1 (PMC13548612; doi:10.1186/s12864-026-13110-1)
Supplement: Supplementary file 1 [file 12864_2026_13110_MOESM1_ESM.docx]

**Additional Files**

**Tables**

**Additional file 1**: Dietary and nutritional composition of diets containing increasing levels of microalgae oil (*Schizochytrium sp*.), expressed on a dry matter (DM) basis.

|  | **Control (n=6)** | **Low**  **(n=4)** | **Medium (n=5)** | **High**  **(n=5)** |
| --- | --- | --- | --- | --- |
| Microalgae oil (g/kg DM) | 0 | 5.4 | 10.8 | 16.2 |
| Soya oil (g/kg DM) | 16.2 | 10.8 | 5.4 | 0 |
| Wheat (g/kg DM) | 98.04 | 98.04 | 98.04 | 98.04 |
| Concentrate meal*(g/kg DM) | 300 | 300 | 300 | 300 |
| Soyabean meal (g/kg DM) | 80 | 80 | 80 | 80 |
| Silage (g/kg DM) | 500 | 500 | 500 | 500 |
| Diet Metabolizable energy (MJ/kg DM) | 11.72 | 11.72 | 11.72 | 11.72 |
| Diet Metabolizable protein (gN/kg DM) | 13.66 | 13.66 | 13.66 | 13.66 |

*at 16% Crude Protein

**Additional file 2**: Total reads, mapping quality and features assigned for the twenty lambs in the study.

| **Animal ID** | **Percent of Assigned features** | **Percent of reads aligned** | **Total Reads in Millions** |
| --- | --- | --- | --- |
| 6764 | 75.00% | 93.40% | 35 |
| 6768 | 76.10% | 93.50% | 33.1 |
| 6856 | 76.20% | 93.20% | 34 |
| 6865 | 73.40% | 92.80% | 32.6 |
| 6914 | 76.70% | 93.10% | 32.9 |
| 6968 | 77.50% | 92.90% | 32.5 |
| 6977 | 69.90% | 91.80% | 27.2 |
| 7025 | 73.40% | 93.80% | 29 |
| 7033 | 63.40% | 81.80% | 28.7 |
| 7050 | 69.40% | 87.60% | 31.2 |
| 7059 | 73.30% | 94.20% | 29 |
| 7066 | 74.30% | 94.40% | 25.6 |
| 7070 | 63.50% | 81.70% | 25 |
| 7093 | 68.90% | 87.40% | 28 |
| 7105 | 71.60% | 93.50% | 24.4 |
| 7115 | 71.30% | 93.20% | 27.6 |
| 7133 | 71.60% | 92.10% | 30.3 |
| 7198 | 72.50% | 92.90% | 27.4 |
| 7220 | 72.80% | 93.20% | 30.6 |
| 7236 | 72.40% | 93.10% | 31.2 |

**Additional file 3**: Differentially expressed genes (DEGs) associated with increasing levels of microalgae oil intake in twenty lambs. The significant threshold was determined as *P_adjusted_* < 0.05 (calculated using Benjamini-Hochberg (BH) method), and absolute |Log_2_ fold change| = 2 with microalgae oil intake treated as a continuous phenotype.

| **Gene** | **baseMean** | **Log_2_FoldChange** | **Raw *P*** | **P_adjusted_** |
| --- | --- | --- | --- | --- |
| *LOC121818748* | 149.03 | -2.92 | 0.0002 | 0.02 |
| *DHCR24* | 2519.07 | -2.91 | 0.0000 | 0.00 |
| *PTGFRN* | 132.78 | -2.88 | 0.0000 | 0.00 |
| *CD101* | 1320.79 | -3.33 | 0.0000 | 0.01 |
| *FCER1G* | 2969.76 | -2.11 | 0.0003 | 0.03 |
| *MPZL1* | 254.81 | 2.30 | 0.0003 | 0.03 |
| *LOC132658556* | 398.18 | 2.26 | 0.0003 | 0.03 |
| *LSS* | 520.69 | -3.40 | 0.0000 | 0.00 |
| *ACSL3* | 738.29 | -2.14 | 0.0000 | 0.00 |
| *LOC132659352* | 124.81 | 3.59 | 0.0000 | 0.00 |
| *PLPP7* | 140.23 | -2.06 | 0.0011 | 0.05 |
| *IER5L* | 729.81 | -3.24 | 0.0002 | 0.02 |
| *LOC114113871* | 588.43 | 2.27 | 0.0012 | 0.05 |
| *LOC121818999* | 188.30 | 2.32 | 0.0001 | 0.02 |
| *CAPN13* | 105.43 | 5.24 | 0.0000 | 0.00 |
| *LOC132659401* | 101.75 | 2.56 | 0.0003 | 0.03 |
| *LOC121819379* | 352.84 | 2.32 | 0.0006 | 0.03 |
| *GNG11* | 290.53 | -2.83 | 0.0008 | 0.04 |
| *LOC101113060* | 121.11 | -2.20 | 0.0002 | 0.02 |
| *INSIG1* | 426.90 | -3.73 | 0.0000 | 0.00 |
| *LDLR* | 371.89 | -4.49 | 0.0000 | 0.00 |
| *CFD* | 1648.12 | -2.40 | 0.0002 | 0.02 |
| *CD14* | 481.57 | -2.29 | 0.0006 | 0.04 |
| *CSF1R* | 3980.83 | -2.72 | 0.0000 | 0.00 |
| *NDRG2* | 252.10 | -2.15 | 0.0011 | 0.05 |
| *LOC101114959* | 984.50 | -2.10 | 0.0010 | 0.05 |
| *HTR1B* | 180.24 | -2.94 | 0.0011 | 0.05 |
| *SQLE* | 484.23 | -3.19 | 0.0000 | 0.00 |
| *LOC101115135* | 123.46 | -2.54 | 0.0000 | 0.01 |
| *LOC114116927* | 82.47 | -2.80 | 0.0004 | 0.03 |
| *CCL5* | 1052.47 | -2.90 | 0.0000 | 0.01 |
| *CXCL16* | 86.83 | -2.26 | 0.0005 | 0.03 |
| *SREBF1* | 751.59 | -2.71 | 0.0000 | 0.00 |
| *RAMP2* | 141.58 | -3.77 | 0.0002 | 0.02 |
| *MRC2* | 258.14 | -2.25 | 0.0002 | 0.02 |
| *LOC121820650* | 91.66 | -2.05 | 0.0002 | 0.02 |
| *LOC105610551* | 120.50 | 2.38 | 0.0000 | 0.01 |
| *TNFRSF8* | 162.48 | -2.80 | 0.0003 | 0.03 |
| *CEBPB* | 3196.17 | -2.55 | 0.0008 | 0.04 |
| *LOC132657778* | 109.67 | -3.70 | 0.0008 | 0.04 |
| *LOC114118069* | 1678.71 | -2.55 | 0.0007 | 0.04 |
| *LOC132657867* | 1467.96 | 2.48 | 0.0004 | 0.03 |
| *NCR3LG1* | 164.09 | -2.35 | 0.0000 | 0.00 |
| *LOC101119159* | 681.62 | 2.15 | 0.0000 | 0.00 |
| *LOC132657848* | 107.78 | -2.41 | 0.0002 | 0.02 |
| *SH3PXD2B* | 96.60 | -2.40 | 0.0001 | 0.02 |
| *HMGCS1* | 1698.00 | -2.27 | 0.0000 | 0.00 |
| *MSMO1* | 502.61 | -2.07 | 0.0000 | 0.00 |
| *SYNM* | 107.37 | -2.47 | 0.0006 | 0.04 |
| *LOC105605761* | 940.45 | -2.06 | 0.0005 | 0.03 |
| *LOC101122983* | 404.08 | -2.23 | 0.0006 | 0.03 |
| *CCR3* | 502.79 | -3.20 | 0.0000 | 0.00 |
| *DAAM2* | 259.26 | -3.01 | 0.0003 | 0.03 |
| *LOC114110105* | 614.76 | 2.62 | 0.0008 | 0.04 |
| *DHCR7* | 526.86 | -2.90 | 0.0000 | 0.00 |
| *KCNQ1* | 97.12 | -2.75 | 0.0002 | 0.02 |
| *SCD* | 325.34 | -4.82 | 0.0000 | 0.00 |
| *LOC114110263* | 425.97 | -2.89 | 0.0005 | 0.03 |
| *MOCOS* | 94.92 | -2.58 | 0.0002 | 0.02 |
| *CASTOR2* | 1091.97 | -2.72 | 0.0000 | 0.01 |
| *HIP1* | 388.57 | -3.46 | 0.0001 | 0.02 |
| *PSD3* | 299.20 | -2.79 | 0.0004 | 0.03 |
| *PCYT1B* | 94.45 | -2.74 | 0.0000 | 0.01 |
| *LOC101114495_1* | 105.21 | -7.13 | 0.0003 | 0.02 |

**Additional file 4**: Pathway enrichments associated with increasing microalgae oil intake. Gene ontology (GO) and Kyoto encyclopedia of genes and genomes (KEGG) enriched terms on 64 DEGs associated with increasing levels of microalgae oil to replace soya oil were identified by over-representation analysis (ORA).

| **GO Terms** | | | | | | | | | | | | | |
| --- | --- | --- | --- | --- | --- | --- | --- | --- | --- | --- | --- | --- | --- |
| **Ontology** | | **ID** | | **Description** | | **Raw *P*** | | ***P*_adjusted_** | | **qvalue** | | **geneID** | |
| BP | | GO:0016125 | | sterol metabolic process | | 2.83722488235043e-05 | | 0.002355471 | | 0.001990707 | | HMGCS1/INSIG1/SQLE | |
| BP | | GO:0006694 | | steroid biosynthetic process | | 3.39731379326536e-05 | | 0.002355471 | | 0.001990707 | | HMGCS1/INSIG1/SQLE | |
| BP | | GO:0008610 | | lipid biosynthetic process | | 1.62878708196174e-05 | | 0.002355471 | | 0.001990707 | | HMGCS1/INSIG1/MSMO1/SCD/SQLE | |
| BP | | GO:0008202 | | steroid metabolic process | | 0.000133472 | | 0.006940546 | | 0.005865745 | | HMGCS1/INSIG1/SQLE | |
| BP | | GO:1901617 | | organic hydroxy compound biosynthetic process | | 0.000181951 | | 0.007569161 | | 0.006397013 | | HMGCS1/INSIG1/SQLE | |
| BP | | GO:0006629 | | lipid metabolic process | | 0.000503232 | | 0.017445359 | | 0.014743801 | | HMGCS1/INSIG1/MSMO1/SCD/SQLE | |
| BP | | GO:0008203 | | cholesterol metabolic process | | 0.001193989 | | 0.031771906 | | 0.026851763 | | HMGCS1/INSIG1 | |
| BP | | GO:1902652 | | secondary alcohol metabolic process | | 0.001374746 | | 0.031771906 | | 0.026851763 | | HMGCS1/INSIG1 | |
| BP | | GO:1901615 | | organic hydroxy compound metabolic process | | 0.001365022 | | 0.031771906 | | 0.026851763 | | HMGCS1/INSIG1/SQLE | |
| BP | | GO:0006633 | | fatty acid biosynthetic process | | 0.002219866 | | 0.041975655 | | 0.035475377 | | INSIG1/SCD | |
| BP | | GO:0046165 | | alcohol biosynthetic process | | 0.002219866 | | 0.041975655 | | 0.035475377 | | HMGCS1/INSIG1 | |
| **KEGG Terms** | | | | | | | | | | | | | |
| **KEGG ID** | **Sub-category** | | **Description** | | **Raw *P*** | | ***P*_adjusted_** | | **qvalue** | | **geneID** | |  |
| oas00100 | | Lipid metabolism | | Steroid biosynthesis | | 3.46815738982021e-08 | | 3.53752053761661e-06 | | 3.35863663014168e-06 | | 101123162/101118320/101106995/101121628/100125351 | |

BP: biological process, MF: molecular function

**Additional file 5**: Pathway enrichments associated with increasing CH_4_ emissions. Gene ontology (GO) and Kyoto encyclopedia of genes and genomes (KEGG) enriched terms on seven differentially expressed methane-associated genes identified by over-representation analysis (ORA).

| **GO terms** | | | | | | |
| --- | --- | --- | --- | --- | --- | --- |
| Ontology | **ID** | **Description** | **Raw *P*** | ***P*_adjusted_** | **qvalue** | **geneID** |
| BP | GO:0009145 | purine nucleoside triphosphate biosynthetic process | 0.003193 | 0.032841 | 0.010563 | NME4 |
| BP | GO:0009201 | ribonucleoside triphosphate biosynthetic process | 0.003193 | 0.032841 | 0.010563 | NME4 |
| BP | GO:0072527 | pyrimidine-containing compound metabolic process | 0.003193 | 0.032841 | 0.010563 | NME4 |
| BP | GO:0009142 | nucleoside triphosphate biosynthetic process | 0.004151 | 0.032841 | 0.010563 | NME4 |
| BP | GO:0009205 | purine ribonucleoside triphosphate metabolic process | 0.005747 | 0.032841 | 0.010563 | NME4 |
| BP | GO:0009199 | ribonucleoside triphosphate metabolic process | 0.006066 | 0.032841 | 0.010563 | NME4 |
| BP | GO:0009144 | purine nucleoside triphosphate metabolic process | 0.006386 | 0.032841 | 0.010563 | NME4 |
| BP | GO:0009141 | nucleoside triphosphate metabolic process | 0.007663 | 0.034483 | 0.011091 | NME4 |
| BP | GO:0009152 | purine ribonucleotide biosynthetic process | 0.011175 | 0.036015 | 0.011584 | NME4 |
| BP | GO:0009260 | ribonucleotide biosynthetic process | 0.012452 | 0.036015 | 0.011584 | NME4 |
| BP | GO:0006164 | purine nucleotide biosynthetic process | 0.012771 | 0.036015 | 0.011584 | NME4 |
| BP | GO:0046390 | ribose phosphate biosynthetic process | 0.012771 | 0.036015 | 0.011584 | NME4 |
| BP | GO:0072522 | purine-containing compound biosynthetic process | 0.01341 | 0.036015 | 0.011584 | NME4 |
| BP | GO:0009165 | nucleotide biosynthetic process | 0.015006 | 0.036015 | 0.011584 | NME4 |
| BP | GO:1901293 | nucleoside phosphate biosynthetic process | 0.015006 | 0.036015 | 0.011584 | NME4 |
| BP | GO:0009150 | purine ribonucleotide metabolic process | 0.018199 | 0.039591 | 0.012734 | NME4 |
| BP | GO:0009259 | ribonucleotide metabolic process | 0.019476 | 0.039591 | 0.012734 | NME4 |
| BP | GO:0019693 | ribose phosphate metabolic process | 0.019796 | 0.039591 | 0.012734 | NME4 |
| BP | GO:0006163 | purine nucleotide metabolic process | 0.023946 | 0.044828 | 0.014418 | NME4 |
| BP | GO:0072521 | purine-containing compound metabolic process | 0.024904 | 0.044828 | 0.014418 | NME4 |
| BP | GO:0009117 | nucleotide metabolic process | 0.028416 | 0.046977 | 0.015109 | NME4 |
| BP | GO:0006753 | nucleoside phosphate metabolic process | 0.029055 | 0.046977 | 0.015109 | NME4 |
| BP | GO:0090407 | organophosphate biosynthetic process | 0.030013 | 0.046977 | 0.015109 | NME4 |
| BP | GO:0055086 | nucleobase-containing small molecule metabolic process | 0.033206 | 0.049808 | 0.01602 | NME4 |
| MF | GO:0016776 | phosphotransferase activity, phosphate group as acceptor | 0.005811 | 0.031372 | 0.025685 | NME4 |
| MF | GO:0019205 | nucleobase-containing compound kinase activity | 0.006972 | 0.031372 | 0.025685 | NME4 |
| **KEGG Terms** | | | | | | |
| KEGG ID | **Sub-category** | **Description** | **Raw *P*** | ***P*_adjusted_** | **qvalue** | **geneID** |
| oas00240 | Nucleotide metabolism | Pyrimidine metabolism | 0.014175 | 0.042138 | NA | NME4 |
| oas00983 | Xenobiotics biodegradation and metabolism | Drug metabolism - other enzymes | 0.016039 | 0.042138 | NA | NME4 |
| oas01232 | Global and overview maps | Nucleotide metabolism | 0.021888 | 0.042138 | NA | NME4 |
| oas00230 | Nucleotide metabolism | Purine metabolism | 0.033797 | 0.042138 | NA | NME4 |
| oas01240 | Global and overview maps | Biosynthesis of cofactors | 0.035115 | 0.042138 | NA | NME4 |
| oas04360 | Development and regeneration | Axon guidance | 0.043534 | 0.043534 | NA | PLXNB3 |

BP: biological process, MF: molecular function

**Figures**

**Fig S1**: Protein-protein interaction (PPI) network of DEGs significantly associated with microalgae oil intake in lambs. The PPI network was constructed using an interaction score cutoff greater than 0.7 reflecting high confidence interactions between the genes.

**Fig S2**: Scatter plots representing CH_4_ emission variations and normalized gene expression levels of seven CH_4_-associated DEGs. The grey area near the red line indicates the standard error of the regression.

**Fig S3**: Heatmap showing gene semantic similarities between microalgae oil intake-associated DEGs with absolute Log_2_ fold change greater than 2 and *P_adjusted_* < 0.05. Semantic similarity scores were generated based on a) cellular component (CC), b) molecular function (MF) and c) biological process (BP) gene ontology categories. Red colour represents higher semantic similarity, and blue represents low similarity between genes.

**Fig S4** Heatmap showing gene semantic similarities between CH_4_-associated DEGs with mapped human ortholog annotations. Semantic similarity scores were generated based on a) cellular component (CC), b) molecular function (MF) and c) biological process (BP) gene ontology domains. Only three genes (*NME4, MARCHF3, PLXNB3*) had human ortholog annotations. Red colour represents higher semantic similarity, and blue represents low similarity between genes.

**Fig S5**: Heatmap showing gene semantic similarities between CH_4_-associated DEGs with absolute Log_2_ foldchange greater than 0 and *P_adjusted_* < 0.05. Semantic similarity scores were generated based on a) cellular component (CC), b) molecular function (MF) and c) biological process (BP) gene ontology domains. MF ontology showed many genes with semantic similarity scores of 1 (red cells in the heatmap) reflecting shared broad biochemical activities.
